# An Early-Onset Advanced Rectal Cancer Patient With Increased KRAS Gene Copy Number Showed A Primary Resistance to Cetuximab in Combination With Chemotherapy: A Case Report

**DOI:** 10.3389/fonc.2021.755578

**Published:** 2021-11-23

**Authors:** Tian Fang, Tingting Liang, Yizhuo Wang, Haitao Wu, Shuhan Liu, Linying Xie, Zhihao Zhang, Jiaying Liang, Cheng Yao, Yehui Tan, Chang Wang

**Affiliations:** ^1^ Cancer Center, The First Hospital of Jilin University, Changchun, China; ^2^ Bethune Institute of Epigenetic Medicine, The First Hospital of Jilin University, Changchun, China; ^3^ Department of Breast Surgery, The First Hospital of Jilin University, Changchun, China

**Keywords:** KRAS, gene copy number, colorectal cancer, cetuximab, case report, anti-EGFR monoclonal antibody, gene amplification

## Abstract

Mutations in *KRAS* (codon 12/13), *NRAS, BRAF* ^V600E^, and amplification of *ERBB2* and *MET* account for 70–80% of anti-epidermal growth factor receptor (EGFR) monoclonal antibody primary resistance. However, the list of anti-EGFR monoclonal antibody primary resistance biomarkers is still incomplete. Herein, we report a case of wild-type RAS/BRAF metastatic colorectal cancer (CRC) with resistance to anti-EGFR monoclonal antibody and chemotherapy. Initially, mutation detection in postoperative tumor tissue by using amplification-refractory mutation system polymerase chain reaction indicated wild-type RAS/BRAF without point mutations, insertion deletions, or fusion mutations. Therefore, we recommended combined therapy of cetuximab and FOLFIRI after failure of platinum-based adjuvant chemotherapy, but the disease continued to progress. Next generation sequencing analysis of the postoperative tumor tissue revealed that *KRAS* copy number was increased and detected *SMAD4*, *RNF43*, and *PREX2* mutations. This is the first case of advanced CRC with increased copy numbers of *KRAS* resistant to cetuximab and chemotherapy, which results in poor patient survival, and other mutated genes may be associated with the outcomes. Our findings indicate *KRAS* copy number alterations should also be examined, especially with anti-EGFR monoclonal antibody therapy in CRC, since it may be related with the primary resistance to these drugs.

## Introduction

Colorectal cancer (CRC) is one of the most common causes of cancer-related deaths worldwide (1). The mean age of patients with CRC ranges from 49 to 60 years. Early onset of CRC generally refers to the onset in patients younger than 50 years of age at the time of diagnosis. It is characterized by a more advanced stage at diagnosis, poorer cell differentiation, higher prevalence of signet ring cell histology, and a primary tumor located on the left colon or rectum (2).

The combination of biological monoclonal antibodies and chemotherapeutic cytotoxic drugs provides clinical benefits to patients with advanced or metastatic CRC (mCRC) (3). Bevacizumab is an anti-vascular endothelial growth factor monoclonal antibody (MoAb) used as a first-line treatment in RAS- or BRAF-mutated mCRC (4). Cetuximab and panitumumab can promote survival in patients with wild-type RAS and BRAF tumors, which target the epidermal growth factor receptor (EGFR) extracellular domain and subsequently inhibit the mitogen-activated protein kinase signaling pathway. The principal downstream effectors of EGFR activation are the RAS/RAF/MEK/ERK, PI3K/AKT/mTOR, and PLCγ/PKC pathways (5), which is a key regulator of cell proliferation, differentiation, division, and survival, and the metastatic potential of tumor cells (6). Mutations in any of the upstream genes may be transmitted to the protein through transcription or translation, resulting in abnormal activation of the signaling pathway (7). Alterations in *RAS*, *BRAF*, *PIK3CA*, *EGFR*, *PTEN*, and *HER2* are key determinants of resistance to anti-EGFR MoAb therapies (8–10). However, little is known regarding patients harboring *KRAS* amplification, and data on the response to anti-EGFR treatment are lacking.

Here, we present a patient with an initial diagnosis of *KRAS*-amplified locally advanced rectal adenocarcinoma who demonstrated no clinical response to anti-EGFR MoAb and chemotherapy, and the disease progressed rapidly. This case report indicates the potential of increased *KRAS* gene copy numbers (GCN) in primary resistance to anti-EGFR MoAb in patients with CRC.

## Case Report

### Case Presentation

**Figure 1:**
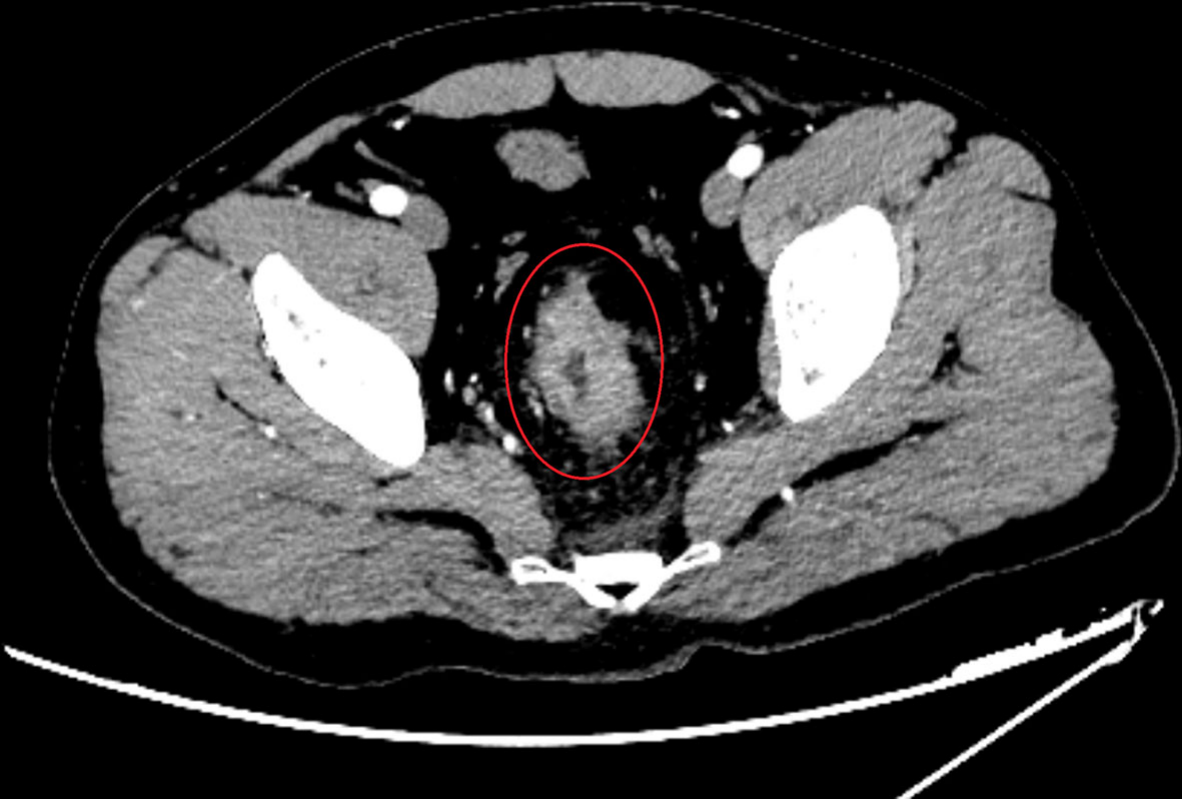
Imaging examinations performed before surgery. Enhanced CT scans on March 21, 2020 of abdomen revealed that occupying lesions in the middle and upper rectum, the intestinal lumen was narrowed, and the serosal layer was hairy. After enhancement, the lesion was uneven and enhanced, and the length of the lesion was about 5.7 cm, considering that was rectal cancer (T4aN1M0).

A 40-year-old man was admitted in our hospital with complaints of diarrhea for one month. Physical examination was normal, but electronic colonoscopy revealed a large polypoid mass in the rectum, situated approximately 9–15 cm from the anal verge. The lesion almost completely occluded the rectal lumen, allowing only a small endoscope to pass through. Pathology revealed an adenocarcinoma. Enhanced computed tomography (CT) of the abdomen revealed occupying lesions in the middle and upper rectum. Considering the possibility of rectal cancer, CT findings were consistent with T4aN1M0 (**Figure 1**). Blood tests showed a slight increase in tumor markers (CA724, 7.79 U/mol; carcinoembryonic antigen, 9.16 ng/mol), creatinine (102.8 μmol/L), direct bilirubin (7.2 μmol/L), and C-reactive protein (13 mg/L). The patient underwent total resection of the rectal cancer and ileostomy on March 24, 2021. After surgery, all blood test results returned to normal.

**Figure 2:**
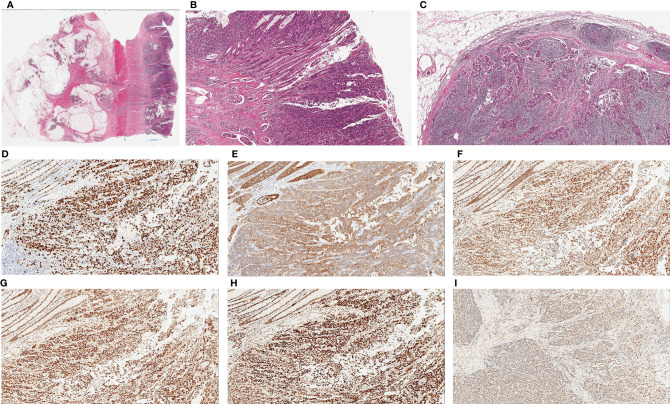
Histopathological examination of tumor. **(A)** Hematoxylin and eosin (H&E) staining of colonic primary tumor shows glandular differentiation and invasion into the entire intestinal wall (×20). **(B)** Poorly differentiated adenocarcinoma on the surface of mucosa with multiple intravascular thrombus in submucosa, (×40). **(C)** Metastatic tumors can be seen in lymph node, (×40). Immunohistochemical staining of tumor cells. Ki-67 partial expression in tumor (+70%) (**D**, ×100), CKpan expression in tumor (**E**, ×100.), Immunostaining for MLH1 (+70%) (**F**, ×100), MSH2 (+70%) (**G**, ×100), MSH6 (+80%) (**H**, ×100)and PMS2 (+50%) (**I**, ×100) confirmed the tumor with proficient MMR.

Surgical pathology findings showed a moderate-to-poorly differentiated adenocarcinoma in the rectum (pT3N2bMx). The tumor volume was approximately 4 cm × 3 cm × 1.2 cm. Cancer infiltration was observed in vessels and nerves. Residual tumors can be detected on the edges of the surgical specimens. Meanwhile, 20 mesenteric lymph nodes were observed, and cancer embolus was distinguished in the local submucosal vessels. Immunohistochemistry results were as follows: pMMR, Ki-67 (+70%); CKpan (+), P53 (−), CgA (−), and Syn (−) (**Figure 2**).

**Figure 3:**
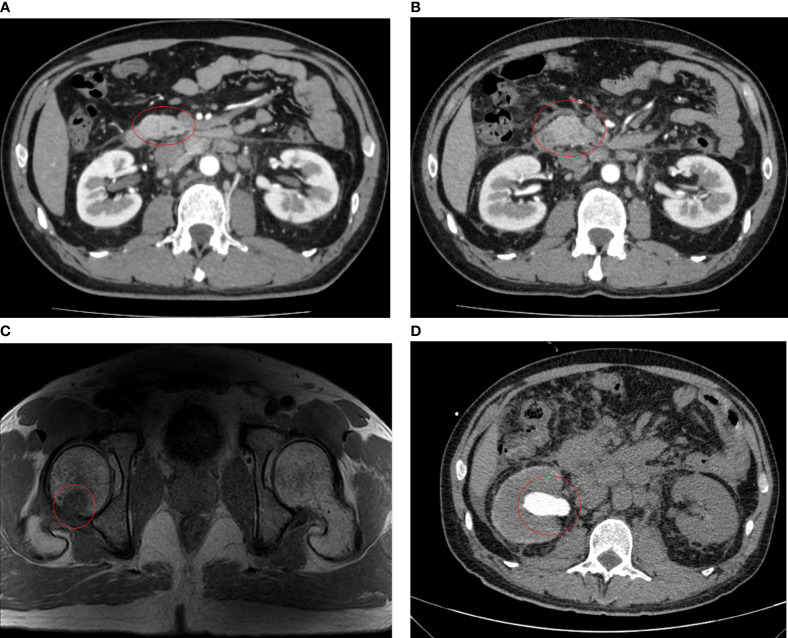
Imaging examinations performed after chemotherapy. **(A)** After five cycles of XELOX, CT scans on August 12, 2020 revealed enlargement of the abdominal para-aortic and bilateral para-iliac lymph nodes, about 0.5–1.5 cm, with slightly enhanced. **(B)** After six cycles of FOLFIRI plus cetuximab treatment, CT scans on November 28, 2020 revealed significant enlargement of lymph nodes in the hilar area, adjacent to the abdominal aorta, and bilateral iliac vessels, with a maximum of about 2.3 cm and slightly enhancement. **(C)** MRI scans on November 29, 2020 show patchy shadows are seen on the right femoral head and greater trochanter, enhanced scans are seen to be enhanced, consider metastasis. **(D)** CT scans on December 22, 2020 showed hydronephrodilation of the right renal pelvis and space-occupying lesion about 16.2 mm × 9.3 mm in the initial segment of the right ureter.

Enhanced pelvic Magnetic Resonance Imaging (MRI) was performed 21 days after the surgery and revealed multiple lymph node shadows with visible enhancement beside bilateral iliac vessels with a short diameter of approximately 0.6–0.7 cm, but postoperative inflammatory changes were not excluded. Therefore, five cycles of adjuvant chemotherapy (XELOX: oxaliplatin, 130 mg/m^2^ on day 1; capecitabine, 1,000 mg/m^2^ twice daily on days 1–14, orally) strategy was recommended from April 14 to July 28, 2020. The CT and MRI evaluations were progressive disease (PD) after chemotherapy, detecting significantly enlarged lymph nodes around the abdominal aorta (0.6-1.5cm) on August 12, 2020 (**Figure 3A**).

Since the first-line platinum-based regiment was unsuccessful, chemotherapy (FOLFIRI: 5-fluorouracil 400 mg/m^2^, IV bolus, folinic acid 400 mg/m^2^, and irinotecan 180 mg/m^2^ on day 1, followed by a continuous 46-h infusion of fluorouracil 2,400 mg/m^2^) in combination with cetuximab (500 mg/m² biweekly on day 1) was administered for six cycles from August 17 to November 12, 2020, considering that the patient harbored no RAS or BRAF alterations according to the mutation profile. After three cycles of chemotherapy and targeted therapy, the patient developed a grade 2 cutaneous toxicity, a single skin impetigo with a diameter greater than 10 mm, and the curative effect was evaluated as stable disease (SD), so we continued the therapy for three more cycles. On November 28, 2020, CT and MRI scans indicated PD, detecting a lymph node around the abdominal aorta enlarged to 2.3 cm (**Figure 3B**), tumor metastases to the right femoral head and greater trochanter (**Figure 3C**), and left cervical lymph nodes enlarged to 11 mm × 8 mm.

Likewise, abdominal CT scans indicated PD again, observing a space-occupying lesion (1.6 cm × 0.9 cm) in the initial segment of the right ureter on December 22, 2020 (**Figure 3D**). Renal function tests suggested that uric acid and creatinine levels continuously increased to 470 µmol/L (normal: 210–430 µmol/L) and 226 µmol/L (normal: 57–97 µmol/L), respectively. The patient decided to stop the treatment and never returned to the hospital after four days of palliative radiotherapy.

### Mutation Analysis

Wild-type *KRAS*, *NRAS*, and *BRAF* were amplified using amplification refractory mutation system polymerase chain reaction (ARMS-PCR) performed on March 20, 2020. Results revealed that the patient had wild-type RAS and BRAF. However, the tumor failed to respond to both treatment options and continued to progress.

Molecular characterization of the blood and postoperative tumor DNA of the patient was performed using next-generation sequencing (NGS) on December 1, 2020. Sequence analyses are shown in **Table 1**. We detected that the patient had mutations in three genes (*SMAD4*, *RNF43*, and *PREX2*), and GCN variations of *KRAS* increased to 7.8 gene copies before chemotherapy. In addition, the mutation abundance of the three mutant genes was observed using blood-based circulating tumor DNA (ctDNA) analysis: *SMAD4* increased from 38.69 to 58.0%, *RNF43* increased from 41.25 to 48.03%, and *PREX2* increased from 39.22 to 52.99%, after the failure of second-line treatment. Likewise, the GCN of *KRAS* also increased to 8.31 gene copies, along with low abundance mutations in the other nine newly confirmed genes—*CTNNB1*, *EPHA5*, *ETV1*, *FANCA*, *JAK2*, *MYC*, *PALB2*, *PIK3CA*, and *SPEN*.

**Table 1:** The results of sequence analyses of paraffin sections and ctDNA on December 1, 2020.

Gene	Mutation site	Mutation abundance (paraffin sections)	Mutation abundance (ctDNA)
**SMAD4**	EXON:2 c.136A>T p.K46*	38.69%	58.0%
**RNF43**	EXON:3 c.354dupC p.C119Lfs*6	41.25%	48.03%
**PREX2**	EXON:28 c.3482G>T p.C1161F	39.22%	52.99%
	EXON:23 c.2558T>G p. L853R	–	1.08%
**SPEN**	EXON:11 c.9239C>T p.P3080L	–	1.66%
**PIK3CA**	EXON:14 c.2115A>C p.Q705H	–	0.69%
**PALB2**	EXON:4 c.212A>G p.E71G	–	1.61%
**MYC**	EXON:3 c.1141A>T p.R381W	–	0.78%
**JAK2**	EXON:9 c.1121G>A p.R374K	–	0.43%
**FANCA**	EXON:35 c.3458A>C p.D1153A	–	0.45%
**ETV1**	EXON:8 c.586T>G p.S196A	–	2.82%
**EPHA5**	EXON:14 c.2363T>C p.M788T	–	0.78%
	EXON:14 c.2446G>C p.V816L	–	0.64%
**CTNNB1**	EXON:3 c.134C>G p.S45C	–	3.76%

### Microsatellite Instability and Tumor Mutation Burden

We analyzed MSI through surgically resected tumor tissues, and the results were reported as microsatellite stability (MSS)/MSI-low. The TMB detected in the tumor tissues was described as low level (2.95 mutations/MB). Furthermore, the TMB detected in ctDNA was reported as medium level (11.82 mutations/MB) after the failure of second-line therapy.

## Discussion

Here, we present a patient with an early onset CRC with an initial diagnosis of moderate-to-poorly differentiated locally advanced rectal adenocarcinoma. Unlike the traditional RAS/BRAF mutant CRCs with poor prognosis, the patient had RAS/BRAF wild-type CRC accompanied with *KRAS* amplification and other less-reported gene mutations. Moreover, the patient’s prognosis was extremely poor, the disease progressed rapidly even with the use cetuximab in combination with chemotherapy. Therefore, we hypothesized that the disease in our patient with early onset CRC was more aggressive due to gene alterations, especially the increase in copy number of *KRAS*. Therefore, we report this case and hope to bring clinical benefits to clinicians.

After the rapid progress of second-line treatment in our case, blood tests and postoperative tumor tissues were performed and analyzed using NGS and retrospective NGS, respectively. Results suggested an increase in *KRAS* copy number before treatment, accompanied by *SMAD4*, *RNF43*, and *PREX2* mutations (mutation abundance was much more than 5%). After treatment failure, blood analysis indicated that the copy number of *KRAS* and clonal mutation abundance of the three genes continued to increase, indicating that the tumor developed from the original clone.

*KRAS* amplification in CRC is a rare event, with an overall prevalence of 0.67–2% (11, 12). Previous studies have identified somatic mutations in *KRAS* as biomarkers for inherent resistance to EGFR-targeted drugs in patients with CRC (13), with a positive tissue mutation rate of 32–52.1% (7). However, alterations are responsive to the anti-EGFR inhibitors cetuximab or panitumumab (11, 14). GCN amplification of *KRAS* leads to the activation of RAS-RAF-ERK or PIK-AKT-mTOR pathways, even without additional activating mutations in the genes (15). In the presence of *KRAS* amplification, cetuximab can partially eliminate the phosphorylation of MEK and ERK but cannot induce growth arrest (13). A retrospective clinical study conducted by Valtorta et al. (14) detected that all four *KRAS*-amplified cases were found among the 53 patients who were resistant to anti-EGFR antibodies, while none of the 44 responders had tumors carrying this molecular alteration. Similarly, tumor biopsies of 10 patients who developed resistance to anti-EGFR showed the emergence of *KRAS* amplification in one case and acquisition of secondary *KRAS* mutations in six cases (13). Another retrospective study by Favazza et al. (11) reported that all eight patients with *KRAS*-amplified mCRC showed disease progression at the time of anti-EGFR therapy, and they concluded that *KRAS* amplification is responsible for the primary resistance to EGFR inhibitors. Meanwhile, *RAS* amplifications (involving *KRAS*, *NRAS*, and *HRAS*) were correlated with a younger median patient age at initial diagnosis and a history of inflammatory bowel disease (11). In the present case, *KRAS* amplification was present before chemotherapy, resulting in resistance to cetuximab. Therefore, patients with advanced mCRC should be monitored not only based on the RAS/BRAF mutation status but also *RAS* amplification, and patients with CRC that do not respond to anti-EGFR MoAbs should be excluded. Moreover, we suggest that genetic testing using NGS should be performed in younger patients to guide decision-making and prolong overall survival, because KRAS amplification is more likely to occur in younger patients.

The mutation frequency and abundance of clonal mutations of *SMAD4*, *RNF43*, and *PREX2* indicated that the tumor was progressing from the original clone. *SMAD4*, *PREX2*, and *RNF43* are involved in the TGF-β, PI3K, and Wnt signaling pathways, respectively. These pathways are involved in cell death resistance, growth suppression, and sustained proliferative signaling, respectively. Several studies have reported chemoresistance and anti-EGFR resistance in patients with *SMAD4*-mutated mCRC (16–18), leading to poor prognosis. Furthermore, *SMAD4* and *RNF43* mutations were more commonly observed in early onset mCRC (≤55 years) (19).

NGS could be considered as the best choice to analyze genetic tests for anti-EGFR therapy because it is superior to ARMS-PCR in copy number detection. Moreover, it saves the amount of samples, is cost- and time-efficient, and has great potential for clinical application to expand testing to include mutations in *RAS* and other less reported CRC-related genes (20). NGS analysis must be carried out in early onset CRCs, family history-related CRCs, and CRC-related cancers to identify cause-effective mutations, elucidate the clinical diagnosis, guide decision-making, and prevent the development of the disease in other family members.

Furthermore, blood-based NGS testing suggested low-abundance mutations (<5%) of nine newly emerged genes, indicating the emergence of tumor subcloning and heterogeneity. These genes have been previously reported to be associated with poor prognosis (21–27), but few reports on the efficacy of chemotherapy or target drugs are available. This suggests that tumors with polygenic mutations tend to be more aggressive in terms of biological behavior, leading to poor survival rates. NGS was partially proven to have a higher sensitivity and specificity for detecting mutations with low abundance than ARMS-PCR. Blood-based NGS testing—ctDNA analysis—offers a convenient way to monitor tumor progression and treatment response. Since tumor mutational profiles are highly variable from person to person, a fixed content panel may be insufficient to track the treatment response in all patients. Compared to tumor tissue biopsies, blood-based ctDNA analyses are minimally invasive and accessible for the regular follow-up of cancer patients. The amount of ctDNA can be quantified, and genetic changes can be identified (28). The ctDNA analysis improves both the specificity and sensitivity of monitoring treatment response across several tumor types. It can identify tumor recurrence potentially earlier than imaging-based diagnosis. When augmented with tumor hotspot genes, it can track acquired drug-related mutations in patients (29).

In conclusion, the present case report identified a rare early onset CRC accompanied by *KRAS* GCN amplification. The disease progressed rapidly, and the effects of chemotherapy and anti-EGFR therapy were poor. This suggests that *KRAS* amplification might be responsible for the primary resistance to anti-EGFR treatment in a small proportion of patients, and targeted drugs should not only be based on *RAS* and *BRAF* mutations in CRCs. Furthermore, NGS has the advantage of discovering genes that affect the efficacy of anti-EGFR therapies, and blood-based serial ctDNA analysis provides a convenient way to monitor the efficacy and resistance mechanism of anti-EGFR MoAbs. With improvements and developments in NGS technology, the identification of copy number variations may provide more implications for the diagnosis and treatment of CRC. Further validation in a larger population is needed to establish a predictive biomarker for resistance to anti-EGFR therapy.

## Data Availability Statement

All data generated or analysed during this study are included in this published article. Additional data and materials related to the genetic tests, pathologic reports, treatment information, and images are available for review upon reasonable request.

## Ethics Statement

Written informed consent was obtained from the patient for the publication of any potentially identifiable images or data included in this manuscript.

## Author Contributions

TF and TL contributed equally to this work. All authors were involved in the drafting of the manuscript. TF, TL, and YW designed the clinical treatment for the patient. ZZ, HW, LX, JL, and CY performed the clinical treatment for the patients. CW and YT provided comments and edited the manuscript to become the final version for submission. All authors contributed to the article and approved the submitted version.

## Funding

This study was supported by Health commission of Jilin Province (NO. 2017J063) and Department of Finance of Jilin Province (NO. JLSWSRCZ2020-0048).

## Conflict of Interest

The authors declare that the research was conducted in the absence of any commercial or financial relationships that could be construed as a potential conflict of interest.

## Publisher’s Note

All claims expressed in this article are solely those of the authors and do not necessarily represent those of their affiliated organizations, or those of the publisher, the editors and the reviewers. Any product that may be evaluated in this article, or claim that may be made by its manufacturer, is not guaranteed or endorsed by the publisher.
